# The Peripheral NK Cell Repertoire after Kidney Transplantation is Modulated by Different Immunosuppressive Drugs

**DOI:** 10.3389/fimmu.2013.00046

**Published:** 2013-02-28

**Authors:** Christine Neudoerfl, Bernadett J. Mueller, Cornelia Blume, Kerstin Daemen, Maja Stevanovic-Meyer, Jana Keil, Frank Lehner, Hermann Haller, Christine S. Falk

**Affiliations:** ^1^Transplant Immunology, Institute of Transplant Immunology, Integrated Research and Treatment Center Transplantation, Hannover Medical SchoolHannover, Germany; ^2^Department for Nephrology and Hypertension, Hannover Medical SchoolHannover, Germany; ^3^Department of Abdominal and Transplant Surgery, Hannover Medical SchoolHannover, Germany

**Keywords:** NK cells, cytotoxicity, cytokine secretion, kidney transplantation, immunosuppression, calcineurin-inhibitors, mTOR inhibitors, donor-specific antibodies

## Abstract

In the context of kidney transplantation, little is known about the involvement of natural killer (NK) cells in the immune reaction leading to either rejection or immunological tolerance under immunosuppression. Therefore, the peripheral NK cell repertoire of patients after kidney transplantation was investigated in order to identify NK cell subsets that may be associated with the individual immune status at the time of their protocol biopsies for histopathological evaluation of the graft. Alterations in the peripheral NK cell repertoire could be correlated to the type of immunosuppression, i.e., calcineurin-inhibitors like Cyclosporin A vs. Tacrolimus with or without addition of mTOR inhibitors. Here, we could demonstrate that the NK cell repertoire in peripheral blood of kidney transplant patients differs significantly from healthy individuals. The presence of donor-specific antibodies was associated with reduced numbers of CD56^dim^ NK cells. Moreover, in patients, down-modulation of CD16 and CD6 on CD56^dim^ NK cells was observed with significant differences between Cyclosporin A- and Tac-treated patients. Tac-treatment was associated with decreased CD69, HLA-DR, and increased CD94/NKG2A expression in CD56^dim^ NK cells indicating that the quality of the immunosuppressive treatment impinges on the peripheral NK cell repertoire. *In vitro* studies with peripheral blood mononuclear cells of healthy donors showed that this modulation of CD16, CD6, CD69, and HLA-DR could also be induced experimentally. The presence of calcineurin or mTOR inhibitors had also functional consequences regarding degranulation and interferon-γ-production against K562 target cells, respectively. In summary, we postulate that the NK cell composition in peripheral blood of kidney transplanted patients represents an important hallmark of the efficacy of immunosuppression and may be even informative for the immune status after transplantation in terms of rejection vs. drug-induced allograft tolerance. Thus, NK cells can serve as sensors for immunosuppression and may be utilized for future strategies of an individualized adjustment of immunosuppression.

## Introduction

Human natural killer (NK) cells are part of the innate immune system and play a critical role in the host defense against pathogens and tumor cells by their cytotoxic potential and the production of cytokines and chemokines. Based on the cell surface density of the CD56 molecule, human peripheral NK cells can be divided into two major subsets (CD56^dim^ and CD56^bright^), with each group displaying distinct phenotypic and functional properties. The markers include the Fcγ-receptor IIIa (CD16), CD6 (Braun et al., 2011), and KIR (killer cell immunoglobulin-like receptors) which are expressed on the majority of CD56^dim^ cells. In contrast to the CD56^dim^ subset, CD56^bright^ NK cells express CD16 and CD6 at very low levels of less than 3% but display a higher density of the inhibitory heterodimer CD94/NKG2A (Yu et al., 2010). Other surface receptors such as the activation-dependent markers CD25, CD69, HLA-DR, and typical NK cell receptors like the natural cytotoxicity receptors (NCR), NKG2D, and CD161 are equally expressed on both subsets. In addition to the distinct phenotypic features, the two NK cell subsets are also associated with different effector functions. It is generally accepted that naïve CD56^dim^ NK cells are primarily responsible for cytotoxic activity due to their high content of granules filled with cytotoxins like perforin, granzyme A, and B. In contrast, CD56^bright^ NK cells are postulated to regulate immune responses primarily through their cytokine production, for example by secretion of interferon (IFN)-γ after stimulation with interleukin (IL)-2, IL-12, or IL-15.

Natural killer cells were recently discussed to be involved in immune recognition after kidney transplantation due to their capability to bind donor-specific anti-HLA-antibodies (donor-specific Ab, DSA) (Farkash and Colvin, 2012). However, the peripheral NK cell repertoire of kidney recipients has not been studied in great detail and the influence of different immunosuppressive regimens like calcineurin (CNI) or mTOR inhibitors on NK cell subset distribution has not been precisely addressed.

Since many years, it is known that DSA have a negative impact on survival and function of kidney transplants (Halloran et al., 1990, 1992; Lucas et al., 2011). Anti-HLA-Ab can be induced in the context of kidney transplantation based on HLA class I- and/or class II mismatches between donor and recipient. Acute antibody-mediated rejection (ABMR) is pathologically characterized by glomerulitis, peritubular capillaritis, dilatation of peritubular capillaries, and interstitial edema (Lefaucheur et al., 2007; Loupy et al., 2009). Acute ABMR occurs within days to weeks after transplantation and is primarily caused by *de novo*-synthesized DSA. Chronic ABMR presents with duplication of the glomerular and/or multilayering of the peritubular capillary basement membrane and possibly with chronic lesions as interstitial fibrosis and tubular atrophy or fibrous intimal thickening of arteries in combination with the presence of anti-HLA-Abs, especially DSA (Liefeldt et al., 2012). One proposed mechanism of these antibodies is activation of complement that can be measured by staining of C4d,  a cleavage product of the activated C4 component of the classical complement pathway (Mengel et al., 2012). Recent work by Terasaki et al. has clearly identified DSA as the major cause of chronic rejection (Terasaki and Cai, 2008; Hidalgo et al., 2010). Since HLA class I- and II-antigens are differentially expressed on immune and non-immune cells, DSA against HLA class I- or II-molecules generate diverse pathological effects in the transplant. While HLA class I specific DSA are more responsible for rapid graft loss, HLA class II specific DSA rather seem to cause chronic rejection (Worthington et al., 2003). Besides HLA-specific antibodies, an involvement of antibodies directed against two polymorphic ligands for the T and NK cell receptor NKG2D, i.e., MICA and MICB, in declining graft function is still controversially discussed (Zou et al., 2007).

The current concept of initial immunosuppression by CNI after kidney transplantation is focused on the suppression of T cell-mediated immune reactions toward the allogeneic organ which is matched for as many as possible HLA class I (A and B) and class II (DR) antigens. Although HLA-C molecules represent prominent KIR ligands, HLA-C typing is not routinely performed and rather ignored along with HLA-DQ and HLA-DP molecules for the allocation of kidneys from deceased donors to renal insufficient patients. The standard immunosuppressive regimen, also at our transplantation unit is based on initial CNI, either cyclosporine A (CsA) or Tacrolimus (Tac) in combination with steroids (prednisolone) and mycophenolate mofetil (MMF) (Heemann et al., 2011). Once treated with this CNI-based regimen, minimization of the drug is associated with a higher risk of rejection. Operational tolerance is defined as a “well-functioning graft lacking histological signs of rejection in the absence of immunosuppressive drugs for at least 1 year in an immune-competent host” (Ashton-Chess et al., 2007; Orlando et al., 2010). Spontaneous tolerance after complete withdrawal of immunosuppression is rare after kidney transplantation. Clinical attempts to induce operational tolerance by different concepts for long-term adaptation of the recipient immune system for an allogeneic organ were not successful so far (Page et al., 2012). Current strategies focus on the identification of tolerance markers under maintained immunosuppression that define a status of “organ acceptance” which supports long-term graft function and survival (Orlando et al., 2010). This clinically relevant “tolerance status” under immunosuppression may be mediated by both adaptive and innate immune cells although this hypothesis has not been proven yet. In tissue biopsies and periphery blood of kidney recipients, NK cell signatures have been recognized in tolerant states (Sagoo et al., 2010) as well as in ABMR (Hidalgo et al., 2010). However, the role of NK cell subsets in this context has not been investigated in detail, especially not in light of different immunosuppressive drugs.

In general, CNI-based immunosuppressive therapies with CsA in combination with steroids and MMF are frequently associated with nephrotoxicity in the graft favoring a development of interstitial fibrosis and tubular atrophy and, on the long run, a decrease in kidney function or even graft loss (Casey and Meier-Kriesche, 2011). Compared to CsA, Tac is suggested to have a lower grade of nephrotoxicity due to its higher immunosuppressive potential even at lower drug concentrations which correlates with lower trough levels that are routinely measured 12 h after medication in serum of kidney Tx patients (Ekberg et al., 2007). This effect is not completely depending on the substance itself but also on interactions with co-medication, e.g., MMF. In Tac-treated patients, effective MMF levels are higher since the depressing effect of cyclosporine on the enterohepatic recirculation of mycophenolate is missing (Kuypers, 2008). Thus, a switch from CsA to Tac represents one possibility to reduce CNI toxicity. Another possibility to minimize nephrotoxicity is an immunosuppressive therapy with mTORi (e.g., sirolimus/Sir or everolimus). Several studies demonstrated that complete withdrawal of CNI after switching to mTORi is clinically safe at least up to 12 months post transplantation (Budde et al., 2011). CNI primarily inhibit T cell activation due to a block in signal transduction at the NFAT-binding protein FKPB12 which prevents transcriptional activation via NFAT and expression of its downstream targets like IL-2, IFN-γ, GM-CSF, and others. In contrast, mTORi block signal transduction of the PI3K/Akt pathway in immune as well as in non-immune cells. While both pathways were studied extensively in T cells as primary target cells of CNI, only limited information is available on the effect of CNI vs. a combination with mTORi on the composition of NK cell subsets in patients after kidney transplantation.

Recent *in vitro* studies have shown differential effects of immunosuppressive drugs on NK cells derived from healthy donors. Treatment of sorted CD56^bright^ and CD56^dim^ NK cells following IL-2 and IL-15 stimulation with immunosuppressive drugs resulted in a more pronounced shift toward the CD56^bright^ subset and KIR/NKG2A expression by the mTORi rapamycin and MPA compared to CsA (Eissens et al., 2010). These phenotypic changes were accompanied by impaired proliferation and cytotoxicity in rapamycin- and MPA-treated NK cells and reduced IFN-γ secretion also by CsA treatment. Similar effects of Tac compared to CsA were demonstrated regarding expansion and phenotypic alterations of IL-2/IL-15-activated purified NK cells of healthy donors (Ohata et al., 2011). These investigations indicate a differential influence of immunosuppressive drugs on the composition of NK cell subsets and their functional status, i.e., cytokine production and cytotoxicity.

In our study, we investigated peripheral NK cell subsets in kidney transplanted patients in correlation to the individual immunosuppressive regimen and the presence of donor-specific antibodies. In addition, we determined the effect of CNI and mTORi on NK cell functions like IL-2-mediated activation, IFN-γ secretion and degranulation *in vitro* in healthy donors. The presence of DSA in kidney recipients was associated with reduced numbers of CD56^dim^ NK cells in peripheral blood. Moreover, the peripheral NK cell repertoire of patients after kidney transplantation was significantly altered compared to healthy donors by a significant decrease in CD16^+^ CD6^+^ CD56^dim^ NK cells. Remarkably, the type of immunosuppression, CsA vs. Tac, had a strong impact on the NK cell phenotype with increased CD69^+^ NK cells in patients under CsA vs. Tac-mediated immunosuppression. These effects in patient blood could also be observed by *in vitro* treatment of NK cells from healthy donors with immunosuppressive drugs. However, it needs to be further investigated whether the loss of CD16 and CD6 expression may represent rather a sign of NK cell activation or functional exhaustion. In summary, we could demonstrate that the NK cell repertoire is altered in kidney recipients according to the individual immunosuppression suggesting that NK cells may serve as sensors of immunosuppression.

## Materials and Methods

### Patients and healthy donors

The collection of blood from patients and healthy donors was approved by the ethics committee at University of Heidelberg, no. S-163/2007, as well as by the ethics committee of Hannover Medical School, no. 968-2011, and kidney recipients gave informed consent on the basis of the ethics vote no. 5970. One hundred twenty-four kidney transplanted patients were enrolled into this study within 2011 and 2012. The cohort had 49 male and 75 female patients, aged 49.56 years ± 14.83 (SD), with a kidney transplant age of 46.19 months ± 16.07 (SD). Regarding immunosuppression, 30 patients received CsA, 78 tacrolimus (Tac group), and 16 tacrolimus in combination with the mTORi, Sir, or everolimus (T/S group). While CsA and Tac-treated patients also receive MMF and steroids (Prednisolon), no MMF is given to patients with low CNI + mTORi (T/S). Peripheral blood mononuclear cells (PBMC) from patients were taken at the time of their protocol biopsy at ≤1 month (*n* = 30), 3 (*n* = 30), 6 (*n* = 24), 9 (*n* = 3), or ≥12 (*n* = 37) months after transplantation. From these 124 patients, 18 were preselected for this NK cell subset analyses based on the histopathological classification of the kidney biopsy at the day of blood withdrawal: unsuspicious histology, T cell-mediated rejection (TCMR), ABMR, and borderline pathology. Regarding immunosuppression, 5 patients were in the CsA, 10 patients in the Tac, and 3 patients in the low-dose CNI + mTORi (T/S) group. Since the patient material is limited by the informed consent, it was not possible to perform these subset analyses with blood from all patients. As normal control samples, PBMC of healthy age-matched volunteers (*n* = 21) where compared to the kidney recipients.

### Phenotypic characterization and absolute numbers of NK cell subsets

Peripheral blood mononuclear cells from patients and healthy donors where separated using Ficoll (Biochrom, Berlin, Germany) separation. The following mAbs were used for cell surface staining by multi-color flow cytometry: CD3-PerCP (clone SK7), CD94-FITC (clone HP3D9), HLA-DR-APC (clone G46-6; BD, Franklin Lakes, NJ, USA), CD56-ECD (clone N901), NKG2A-PE (clone Z199), CD69-PE (clone TP1.55.3), CD25-FITC (clone M-A251), CD16-FITC (clone 3G8; Beckman Coulter, Krefeld, Germany), CD161-PerCP-Cy5.5 (clone HP-3G10, eBioscience, San Diego, CA, USA), and CD6-APC (clone MT411, Miltenyi Biotec, Auburn, CA, USA). PBMC were incubated with mAbs for 30 min at 4°C, washed with PBS (Life Technologies, NY, USA) with 2.5% FBS (Life Technologies) and 0.1% sodium azide (Sigma-Aldrich, St. Louis, MO, USA) and fixed with PBS containing 1% paraformaldehyde (Merck, Darmstadt, Germany). Cells were analyzed by flow cytometry (LSRII, BD), Data were processed using FACS-Diva software 6.1.2 (BD). Absolute lymphocyte numbers (e.g., CD56^+^ NK cells) per μl of peripheral blood where determined via Trucount™ (BD) analysis using 50 μl EDTA blood and fluorochrome-labeled mAb for CD56 (PE) + CD16 (PE) with a known number of beads. Absolute NK cell numbers per μl were calculated by counting CD3^−^ CD56/CD16^+^ events and normalization to beads using the formula: absolute NK cell count per μl = (number of events in cell population/number of events in absolute count bead region) × (number of known beads per test/test volume).

### Identification of allo-HLA, donor-specific, and MICA-specific antibodies

Serum samples were collected at the same points of time as protocol biopsies. The presence of anti-HLA-antibodies as well as MICA antibodies in all patient sera was detected by using the Luminex-based LABScreen-Mixed-Assay (One Lambda, Canoga Park, CA, USA). All samples that were unambiguously positive for anti-HLA class I and/or class II antibodies (MFI > 1000) were further tested for DSA using the LABScreen Single Antigen beads (One Lambda). All tests were performed according to the manufacturer’s guidelines.

### *In vitro* analyses of the effect of CNI, mTORi, and MMF on activation and proliferation, degranulation, and IFN-γ ELISpot assays

The influence of immunosuppressive drugs on NK cell function was analyzed *in vitro* by proliferation, degranulation, and IFN-γ ELISpot assays using PBMC from healthy individuals. For the activation assay, 1 × 10^5^ PBMC were incubated for 4 days in RPMI1640 (Invitrogen) supplemented with 2 mM l-glutamine, 1 mM sodium pyruvate, 100 U/ml penicillin/streptomycin, and 10% fetal calf serum (FCS, Invitrogen) in the presence or absence of IL-2 (500 U/ml) with or without 10 μM of either CsA, Tac, Sir (all three LC Laboratories, Woburn, MA, USA), MMF (Selleckchem, Houston, TX, USA), or equal concentrations of DMSO (Carl Roth, Karlsruhe, Germany). The cells were harvested, washed once in PBS and stained with CD25-FITC, CD69-PE, CD56-ECD, CD3-PerCP, and CD16-Pacific Blue Ab as described above.

CD107a-Degranulation assays were performed with plate bound mAbs against CD16 (clone 3G8, Beckmann Coulter) or the isotype control IgG1 (clone MOPC21, Sigma) in a 96 well U-shaped plate (BD). Goat-anti-mouse-F(ab)_2_ (Beckman Coulter) was coated over night with 2 μg/ml in boracid containing buffer (0.05 M, pH 8.6). After washing twice with PBS, mAbs (10 μg/ml in PBS) were coated for 1 h at 4°C. The plate was washed twice with PBS, 2.5 × 10^5^ PBMC were incubated for 4 h in RPMI 1640 supplemented with 2 mM l-Glutamine, 1 mM Sodium Pyruvate, 100 U/ml Penicillin/Streptomycin, and 10% FCS with or without 10 μM of either of the drugs or the solvent DMSO in the presence of anti-CD107a-PE-Ab (clone H4A3, BD). After 1 h incubation, Monensin (40 μM, Sigma) was added. Cells were harvested after 4 h, once washed in PBS and stained with CD3-PerCP, CD56-ECD, and CD16-FITC Ab as described above.

For the IFN-γ-ELISpot, the “human IFN-γ ELISPOT antibody pair” (detection and capture anti-IFN-γAb, BD) was used. The assay was performed according to the manufacturer’s instructions except for the use of the alkaline phosphatase-streptavidin (BD) for binding to the biotinylated anti-IFN-γ detection Ab. For detection of IFN-γ spots, the AP-conjugated substrate kit (Bio-Rad, Hercules, CA, USA) was used. PBMCs of healthy donors (*n* = 3) were stimulated in the presence of the HLA-negative target cell line K562 with a ratio of 3:1 respectively 1:1 (PBMC cell numbers were donor-dependent either 6/2 × 10^5^ or 3/1 × 10^5^ per well) in triplicates and two titrations as recommended by CIMT. The stimulation was performed for 18 h in RPMI 1640 supplemented with 2 mM l-glutamine, 1 mM sodium pyruvate, 100 U/ml penicillin/streptomycin, and 10% FCS with or without 10 μM of either drug or DMSO solvent.

The number of IFN-γ producing NK cells was determined by capturing IFN-γ to the plate and detection by the biotinylated mAb. Immunospots were quantified by the Immunospot S5 UV-Reader (CTL, Shaker Heights, OH, USA); plates were scanned by Immunospot 5.0-Software (CTL) and the data were analyzed by the Immunospot 5.1 professional software (CTL).

For the proliferation assay, PBMC of four healthy donors were labeled with eFluor 670 (eBioscience, Frankfurt, Germany) according to the manufactures protocol. EFluor 670 binds to cellular proteins and during cell division the dye is distributed equally to the divided cells. 2 × 10^5^ eFluor labeled PBMC where incubated either alone or in the presence of 1 × 10^5^ HLA class I-negative K562 cells (irradiated with 100 Gy) in absence or presence of DMSO, CsA, Tac, Sir, or MMF (each with 10 μM) in a 96 well U-shaped plate (BD). After 48 and 96 h, respectively, cells were harvested, washed once in PBS, and stained with CD3-PerCP and CD56-ECD Ab as described above and analyzed by flow cytometry gating on CD3^−^ CD56^+^ eFluor 670^low^ cells as % of proliferating NK cells.

#### Statistics

All statistical calculations were performed with the GraphPad Prism 5.01 program (La Jolla, CA, USA). The sample values and replicates, respectively, were applied first to three normality tests, Komolgorov–Smirnov, D’Agostino–Pearson, and Shapiro–Wilk followed by either *t*-tests for Gaussian distributed data or Mann–Whitney *U*-tests for non-parametric data sets.

## Results

### The peripheral NK cell repertoire of kidney transplant recipients is influenced by DSA and the type of immunosuppression

**Figure 1:**
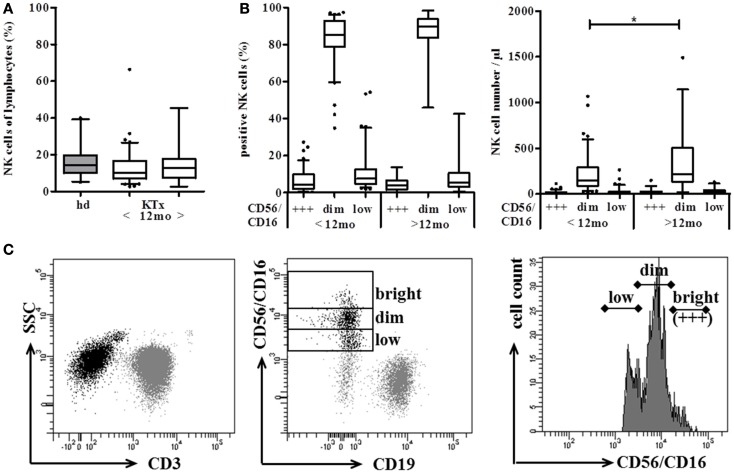
**Distribution of NK cells shows marginal differences between kidney transplanted patients (KTx) and healthy donors**. Proportion and absolute number of CD56^+^ CD3^−^ NK cells were compared between KTx-patients (*n* = 120, white boxes) and healthy donors (*n* = 21, gray boxes) using the standardized Trucount™ FACS analysis. **(A)** KTx-patients were divided into two groups according to the time after transplantation (group 1 < 12 and group 2 > 12 months after Tx) and the percentage of NK cells was compared to healthy donors. **(B)** NK cells of KTx-patients are further subdivided into CD56/16^bright^, CD56/16^dim^, and CD56/16^low^ NK cells using the histogram analysis of the CD16/CD56 PE-staining. NK cells were either calculated as % NK cells of lymphocytes (left) or plotted as absolute NK cell numbers per μl blood (right). **(C)** Gating strategy of the Trucount™ staining procedure: CD45^+^ CD3^−^ lymphocytes (left) were classified into CD56^+^ CD16^+^ NK cells and CD19^+^ B cells (middle). According to their PE-staining, CD56^+^ CD16^+^ NK cells were then further subgrouped into CD56/16^bright^ (+++), CD56/16^dim^, and CD56/16^low^ NK cells (right). The box plots show median values and the whiskers represent the 5–95 percentiles. Asterisks indicate the *p*-values, * is defined as *p* ≤ 0.05, ** as *p* ≤ 0.01, and *** as *p* ≤ 0.001.

The distribution of the two major NK subsets, CD56^dim^, and CD56^bright^ NK cells is rather stable in peripheral blood of healthy volunteers. In order to identify an influence of DSA and the immunosuppressive regimen on NK cells in kidney transplanted patients, the number and subset distribution of NK cells were analyzed in 120 kidney recipients (KTx) at <1, 3, 6, and 12 months (*n* = 86) and >12 months (*n* = 34) after transplantation. Regarding the percentage of NK cells in the lymphocyte population, no significant differences could be observed between KTx-patients either in the group between 1 and 12 months or in the group >12 months after transplantation and healthy volunteers (*n* = 21; Figure 1A). For the determination of NK cell numbers per μl, the Trucount staining procedure was performed in which NK cells are marked by CD56 PE and CD16 PE antibodies in combination with T and B cell markers. This NK cell staining combination was developed for routine FACS staining of whole blood and, thus, cannot be changed without leaving the approved protocol for clinical immunology. In order to discriminate between the CD56^bright^ and CD56^dim^ NK cell subsets despite simultaneous CD16 staining, histogram analyses of the CD16/CD56 PE-staining were performed as indicated by the three boxes in Figure 1C. In healthy donors, the combined CD16/CD56 staining nicely reflected the CD56^dim^ and CD56^bright^ single staining (data not shown). In KTx-patients, an additional NK cell subset with markedly reduced CD16/CD56 density was detectable that was defined as CD56/16^low^ subset (Figure 1C). Thus, NK cells were divided into three subsets, i.e., CD56/16^bright^, CD56/16^dim^, and CD56/16^low^, for all Trucount analyses and the subset distribution as well as the number per μl blood were determined. The CD56/16^low^ NK cell subset was detectable in all KTx-patients and the amount ranged from 1.9 to 54.3% of NK cells (2–263 cells per μl blood, Figure 1B). Since CD56 and CD16 are both stained with PE-labeled mAb in this routine FACS combination, it was not possible to assign this decreased expression to the CD56 or CD16 surface molecule. Therefore, single staining analyses were performed demonstrating that the CD56 histograms displayed the typical CD56^bright^ and CD56^dim^ densities with some individual variation in the proportion of the two subsets. However, the CD16 histograms clearly discriminated between those patients with a high proportion of CD16^+^ NK cells and those patients with CD16 down-regulation. Thus, we hypothesized that the CD56/16^low^ phenotype resulted from decreased CD16 surface expression which was further investigated in detail by a cohort of 18 patients (Figure 4). An influence of the time after KTx on NK cell distribution could be excluded since the division into patients < or >12 months after Tx displayed only minor differences. Only the numbers but not the percentages of CD56/16^dim^ NK cells were slightly higher (*p* < 0.02) in the patient group with a kidney graft for more than 12 months. Thus, long-term treatment with immunosuppressive drugs for more than 1 year is not associated with a manifestation of severe alterations in the NK cell repertoire.

**Figure 2:**
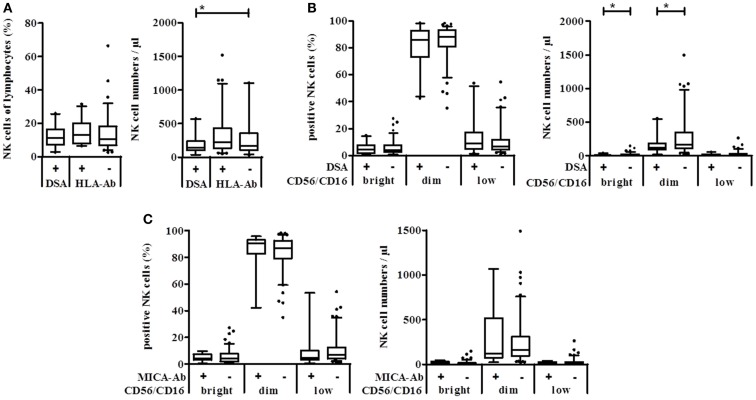
**Natural killer cell numbers correlate with the presence of DSA but not with MICA antibodies in kidney transplanted patients**. Anti-HLA class I, class II, and MICA antibodies were detected in sera of kidney transplanted patients using the Luminex technology. Absolute NK cell numbers and % NK cells of lymphocytes were measured using Trucount™ FACS staining of patient blood samples. **(A)** Percent (left) or absolute number per μl blood (right) of NK cells of patients with DSA, other HLA-specific or no antibodies. **(B)** According to their CD56/CD16 PE-density, NK cells of DSA positive (+) or negative (−) KTx-patients were grouped into CD56/16^bright^, CD56/16^dim^, and CD56/16^low^ NK cells and plotted either as % of lymphocytes or as cell number per μl blood. **(C)** The same analysis was performed with patients grouped according to the presence of MICA-specific Ab.

When patients were grouped according to the presence of DSA, non-organ-specific anti-HLA-antibodies (HLA+), or no HLA antibodies (HLA−) in their serum, no significant differences could be observed in the percentage of NK cells in the lymphocyte gate. However, patients with DSA had significantly lower NK cell numbers compared to patients without any HLA antibodies (Figure 2A). The discrimination into the CD16/CD56 density subsets revealed that only the number but not the percentage of CD56/16^bright^ and CD56/16^dim^ NK cells was significantly decreased in patients with DSA (Figure 2B). Remarkably, the presence of MICA-specific Abs in KTx-patients (*n* = 13) is not associated with a significant modulation of the NK cell subset distribution (Figure 2C) suggesting that only antibodies against allogeneic HLA molecules of the current transplanted organ, i.e., DSA, may have an impact on the peripheral NK repertoire. Whether this decrease in FcγIII-positive NK cells in the periphery is associated with a migration into the allogeneic kidney could not be addressed in this cohort but will be analyzed in detail in future studies.

**Figure 3:**
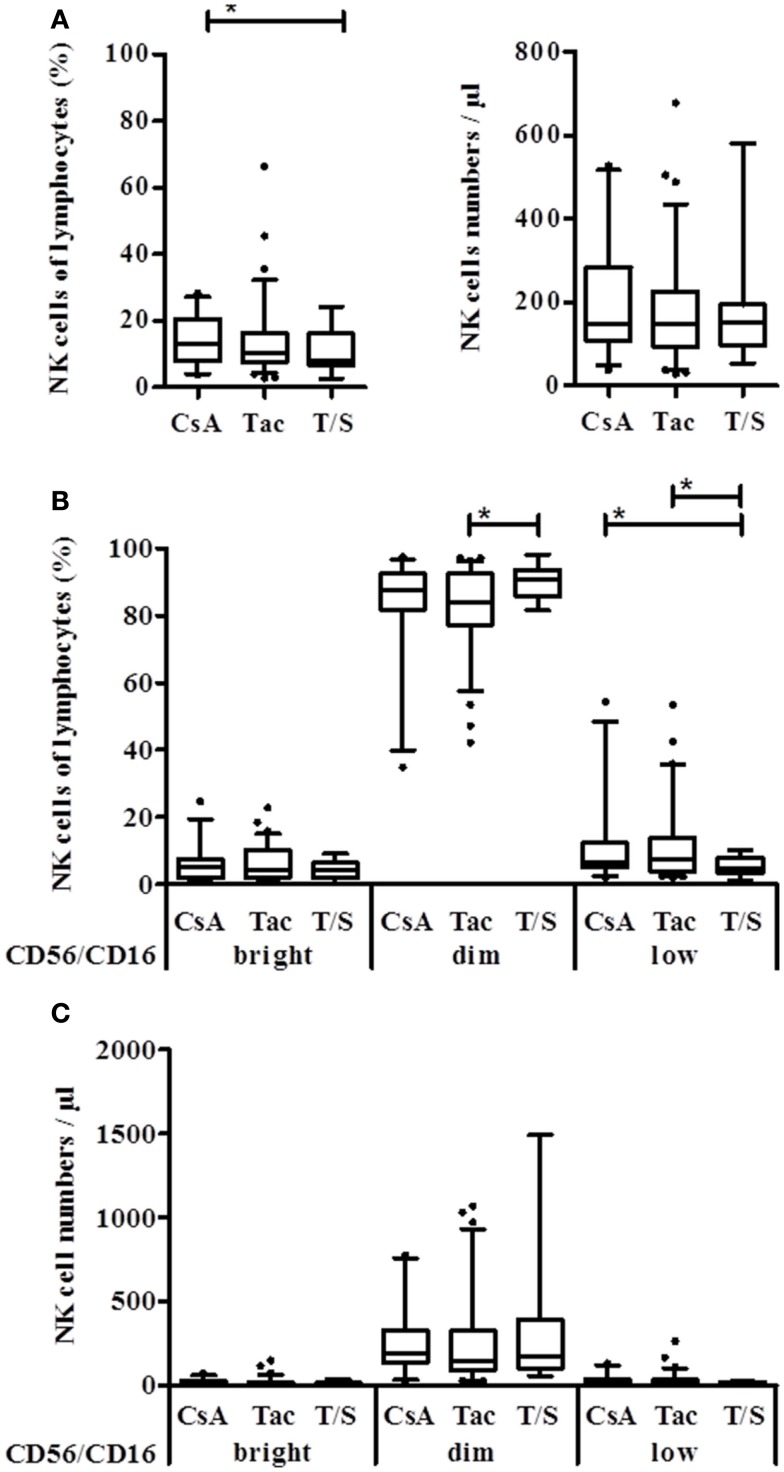
**Natural killer cell distribution of KTx-patients is influenced by different immunosuppressive drugs**. Kidney transplanted patients were grouped according to the immunosuppressive regimen, i.e., CsA, Tac, or combination of Tac + Sir (T/S) and the NK cell proportion of lymphocytes as well as the number per μl blood was compared between the groups. **(A)** Based on their CD16/CD56 PE-staining, NK cells were subdivided into CD56/16^+++^, CD56/16^dim^, and CD56/16^low^ NK cells and calculated as % of lymphocytes **(B)** or absolute numbers per μl **(C)** for each treatment group.

Since the DSA effect was observed in the CD56/16^bright^ and CD56/16^dim^ NK subset, DSA seemed not to be linked to the appearance of the CD56^low^ NK subset in KTx-patients. Therefore, patients were divided into immunosuppressive groups of CsA, Tac, and combined Tac + Sir (T/S) treatment. No significant difference between these groups could be observed regarding absolute NK cell numbers but the percentage of NK cells in the T/S group was significantly lower than in the two other groups (*p* = 0.0376, Figure 3A). In the T/S group, the percentage of CD56/16^dim^ NK cells was significantly higher compared to the Tac group and the percentage of the CD56/16^low^ NK cells was significantly lower in the T/S group compared to the CsA and Tac groups (Figure 3B). Since CD56/16^low^ NK cells are not detectable in healthy donors (data not shown), CsA and Tac seem to be responsible for the appearance of this new CD56/16^low^ NK subset and the addition of an mTOR inhibitor seems to counterbalance this effect. In terms of absolute NK cell numbers, the tendency was the same but no statistical significance could be reached (Figure 3C).

### CsA and Tac differ in their effect on CD16^+^ CD6^+^ NK subsets and the expression of activation markers

**Figure 4:**
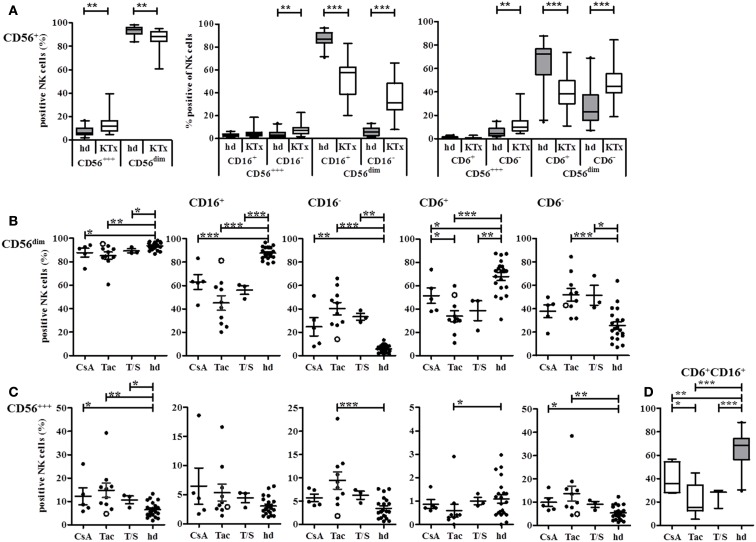
**CD16 and CD6 expression on NK cells is influenced by different immunosuppressive treatments or KTx-patients**. NK cells in PBMC of healthy donors (*n* = 21, gray boxes) or KTx-patients (*n* = 18, white boxes) were analyzed by flow cytometry with individual staining of CD56, CD16, and CD6. **(A)** The proportion of CD56^bright^ and CD56^dim^ NK cells was compared between patients and healthy individuals. **(B)** NK cells were subdivided into CD16^+^ and CD16^−^ or CD6^+^ and CD6^−^ CD56^dim^ NK cells and each subset was compared between patients and healthy individuals. KTx-patients were further classified into three groups regarding their immunosuppressive regimen: CsA, Tac, and combination of Tac + Sir (T/S). **(C)** NK cells were subdivided into CD16^+^ and CD16^−^ or CD6^+^ and CD6^−^ CD56^bright^ NK cells and each subset was compared between patients and healthy individuals. KTx-patients were further classified into three groups regarding their immunosuppressive regimen: CsA, Tac, and combination of Tac + Sir (T/S). One patient was initially treated with CsA for 5 months after KTx and then switched to Tac for the following 7 months [**(B,C)**, open circle]. **(D)** CD6/CD16 double-positive NK cells are calculated for each treatment group and healthy individuals. The combined expression of the different markers is given as percentages of all CD3^−^ CD56^+^ NK cells. Each data point indicates one donor, and median values with standard deviations are depicted as black bars.

These differential effects of CsA vs. Tac and T/S on NK cell subsets became even more pronounced in detailed NK subset analyses using CD16, CD6, CD161, and CD94/NKG2A as NK subset, and CD69, HLA-DR as activation markers. In the comparison of 18 KTx-patients and 21 healthy donors, KTx-patients displayed a significant shift toward the CD56^bright^ NK subset with increased percentages of both CD16^−^ as well as CD16^+^ CD56^bright^ NK cells. The KTx-patients showed significantly deceased amounts of CD16^+^ and CD6^+^ CD56^dim^ NK cells compared to healthy donors in parallel to a significant increase in CD16^−^ and CD6^−^ CD56^dim^ NK cells (Figure 4A). The distribution of 18 patients into three treatment groups clearly identified a treatment-related cause of this down-regulation. Although all KTx patient groups are significantly differed in their NK repertoire compared to healthy individuals, the Tac-treated patient group showed the most significant differences by decreased CD16^+^ and CD6^+^ CD56^dim^ and elevated CD16^−^ and CD6^−^ CD56^dim^ NK cells (Figure 4B). Since CD16 CD6 double-positive CD56^dim^ NK cells represent the major NK subset in peripheral blood, the same strong impact of Tac was also observed for this subset. Treatment with Tac alone was also strongly associated with increased CD16^−^ CD6^−^ CD56^bright^ NK cells (Figure 4C). Even at the very low level of <1.5% CD6^+^ CD56^bright^ NK cells in healthy donors, Tac-treatment in KTx-patients resulted a further decrease of this subset. Interestingly, addition of the mTORi Sirolimus seemed to counterbalance the Tac effect because the values of the T/S group were closer to the CsA group.

The impact of initial immunosuppression directly at KTx could be demonstrated exemplarily by one patient, marked as open circle who was initially treated with CsA for 5 months and switched to Tac for the next 7 months until the date of protocol biopsy and blood withdrawal. Due to his actual immunosuppressive medication by Tac, the patient was assigned into the Tac group. However, the values of his NK cell subsets followed rather the CsA than the Tac pattern indicating that the initial CsA-based modulation of the NK cell repertoire was continued even after the switch to Tac for 7 months.

**Figure 5:**
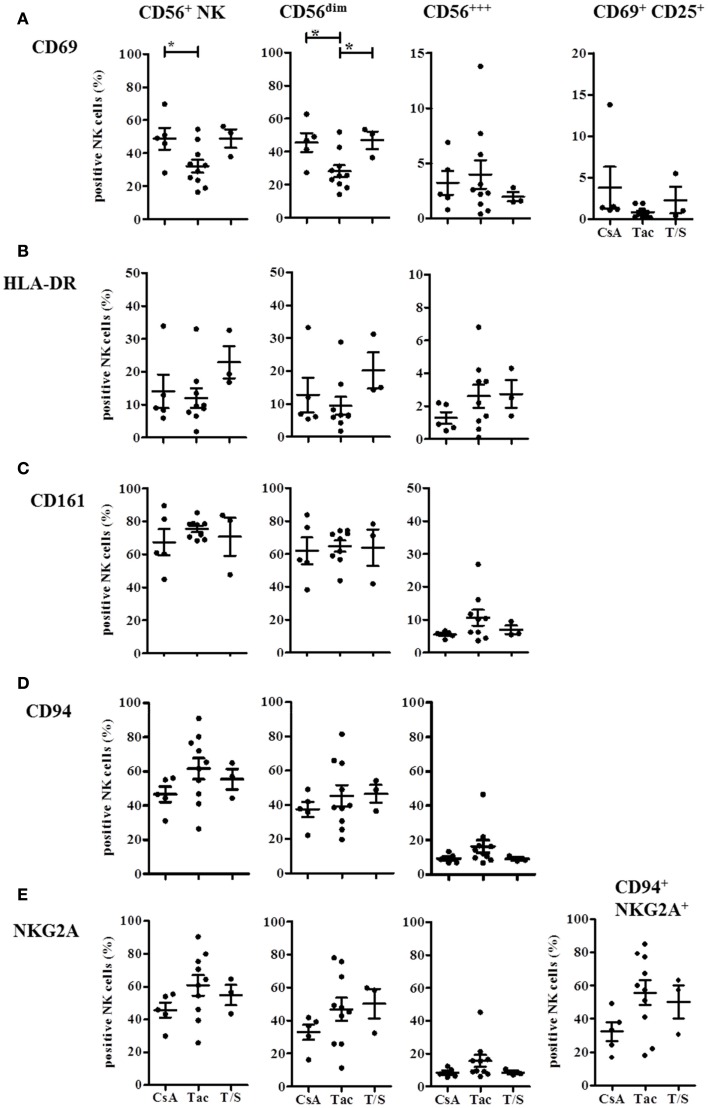
**Immunosuppressive drugs modulate expression of CD69, HLA-DR, CD161, and CD94/NKG2A on CD56^bright^ and CD56^dim^ NK cells in KTx-patients**. KTx-patients (*n* = 18) were grouped according to immunosuppressive drugs (CsA, Tac, and T/S) and the expression of several activation markers was analyzed on total CD56^+^ NK cells and the CD56^bright^ and CD56^dim^ subsets, respectively. CD69 and combined CD69/CD25-expression is shown in **(A)**, HLA-DR expression in **(B)**, CD161 expression in **(C)**, CD94 single expression in **(D)**, and NKG2A single and CD94/NKG2A heterodimer expression in **(E)**. The single or combined expression of these different markers is given as percentage of all CD3^−^ CD56^+^ NK cells.

The detailed analysis of the activation markers CD69, HLA-DR, and CD25 revealed that both CD69 and HLA-DR were decreased on CD56^dim^ NK cells in the Tac group compared to the CsA group although only CD69 reached statistical significance (Figures 5A,B). The opposite trend was observed with the T and NK cell marker CD161 that was expressed by almost all NK cells in the Tac group (Figure 5C) with the highest density on CD56^bright^ NK cells (data not shown). This elevated expression in the Tac group compared to CsA and T/S groups was also detected for the inhibitory heterodimer CD94/NKG2A (Figures 5D,E). Since in healthy donors, virtually all NK cells, CD56^bright^ NK cells in particular, express CD94 and most of them the CD94/NKG2A heterodimer, the low amount of CD94/NKG2A expressing CD56^dim^ NK cells in the CsA group indicates that in parallel to the phenotypic alterations, the balance between activating and inhibitory receptors is also influenced by the immunosuppressive treatment. Despite high proportions of CD69 and HLA-DR positive NK cells, CD25 was only detectable at very low levels on NK cells of these KTx-patients with <3% positive cells. Therefore, it seems to be unlikely that the NK cells in KTx-patients have undergone recent activation because this would be detectable by CD25 co-expression.

These phenotypic analyses indicate that immunosuppression with both CNI and mTORi promotes CD56^bright^ NK cells and causes a shift toward the CD16^−^ CD6^−^ CD56^dim^ NK cell subset. While CsA treatment seemed to be associated with higher proportions of CD69^+^ HLA-DR^+^ NK cells and lower CD161, CD94/NKG2A expression, Tac-treatment seemed to support an opposite phenotype of CD161^hi^, CD94/NKG2A^hi^, CD69^lo^, HLA-DR^lo^ NK cells.

### CsA, Tac, and mTOR inhibitors have differential effects on NK cell activation, degranulation, proliferation, and IFN-γ secretion *in vitro*

**Figure 6:**
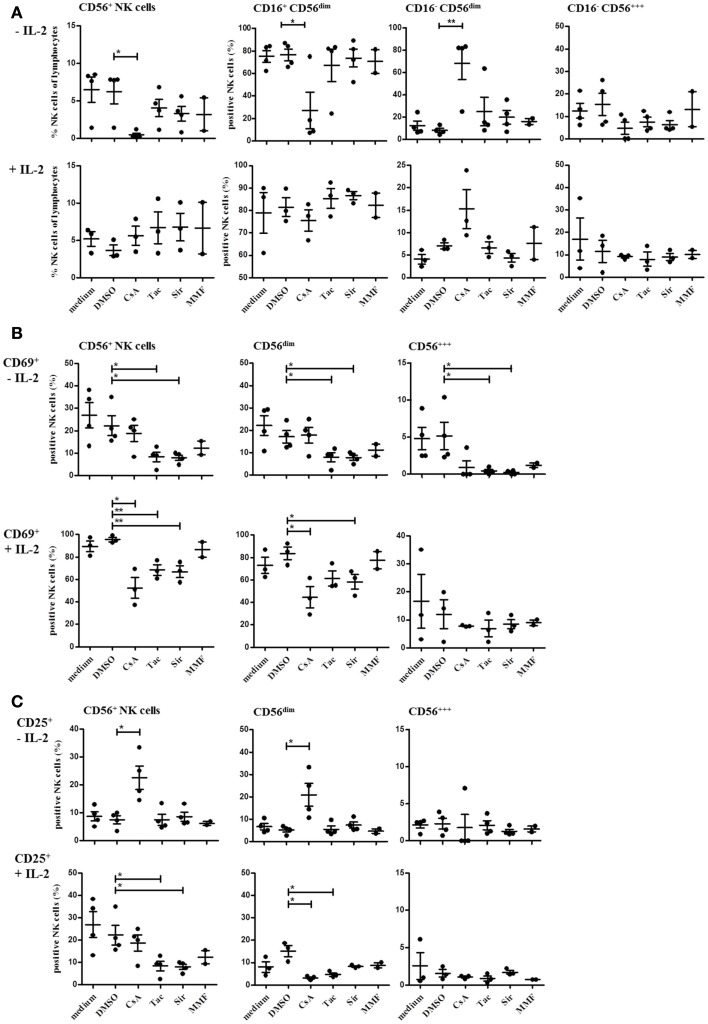
**Natural killer cell activation is differentially modulated by treatment with CsA, Tac, Sir, or MMF**. PBMC of three or four healthy donors were incubated for 4 days with or without 500 U/ml IL-2 in the presence CNI (CsA and Tac), mTORi (Sir), or mycophenolate (MMF) at a concentration of 10 μM, DMSO control solvent or medium. The percentage of all NK cells (CD3^−^/CD56^+^) and of the major NK cell subsets defined as CD16^+^/CD16^−^ CD56^dim^ and CD56^bright^ **(A)** as well as CD69^+^ **(B)** and CD25^+^ NK cells **(C)**were determined by flow cytometry. Each data point indicates one donor, and the median values with standard deviations are depicted as black bars.

Since the differences in NK cell repertoires between the treatment groups were observed in patients at 3, 6, or 12 months after transplantation, we wanted to investigate whether the effects of the two CNI, the mTORi, and MMF are already detectable *in vitro* after several hours or days. Therefore, we performed a number of *in vitro* experiments with PBMC of healthy donors that were activated in the presence of the different immunosuppressive drugs, i.e., CsA, Tac, the mTORi Sir and MMF, or the solvent DMSO, respectively. NK cell activation after 4 day culture in the presence or absence of IL-2 (500 U/ml, 4 days) was analyzed under the influence of the different immunosuppressive drugs (Figure 6). In all donors (*n* = 4), CsA treatment without IL-2 stimulation resulted in a stronger decrease in NK cells compared to Tac, Sir, and MMF that affected primarily the CD16^+^ CD56^dim^ and the CD56^bright^ NK cell subsets. The few remaining NK cells showed a CD16^+^ CD56^bright^ phenotype. Simultaneous stimulation with IL-2 completely prevented this NK cell depletion which was partially caused by induction of apoptosis (not shown). Differences between the drugs became more obvious in the direct comparison of the activation markers CD69 and CD25 (Figure 6B). In the absence of IL-2 stimulation, treatment with Tac and Sir but not CsA and MMF resulted in significantly reduced CD69 expression on both CD56^bright^ and CD56^dim^ NK subsets. Following IL-2 stimulation, almost all NK cells expressed CD69 after 4 days in medium and DMSO control samples. However, treatment with CsA, Tac, and Sir but not with MMF significantly inhibited the CD69 up-regulation primarily on CD56^dim^ NK cells (Figure 6B). The CD25 induction by IL-2 seems to involve different mechanisms because in the absence of IL-2, CsA alone was able to mediate CD25-expression on the few remaining CD56^dim^ NK cells. Following IL-2 stimulation, CsA, Tac, and Sir significantly prevented CD25 induction on CD56^dim^ NK cells in contrast to MMF that had only minor effects on both NK subsets (Figure 6C). Thus, simultaneous IL-2 receptor-mediated signals interfere with both inhibitor classes, CNI and mTORi, but not with the metabolic inhibitor MMF.

**Figure 7:**
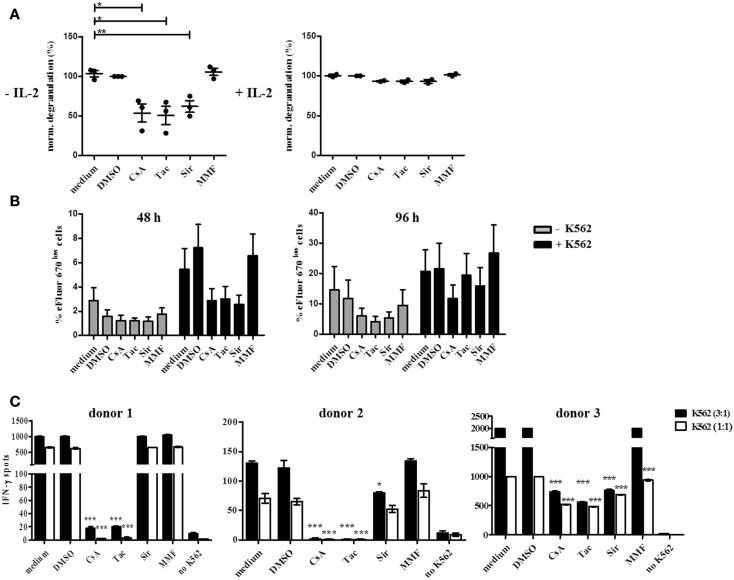
**Natural killer cell degranulation, proliferation, and IFN-γ production is differentially inhibited by CsA, Tac, Sir, or MMF treatment and this effect is counterbalanced by IL-2**. **(A)** Resting (−IL-2, *n* = 3) or 48 h pre-activated PBMC (500 U/ml IL-2, *n* = 2) of healthy donors were stimulated by plate bound CD16 Ab in the presence of CsA, mTORi, or MMF (10 μM), DMSO control solvent or medium. Degranulation was measured after 4 h by CD107a-staining of CD3^−^ CD56^+^ NK cells. Baseline degranulation with isotype Ab reached <5% of resting and <26% of IL-2 activated NK cells (data not shown). **(B)** eFluor 670 stained PBMC of healthy donors (*n* = 4) were either cultured alone or in the presence of irradiated K562 cells for 48 or 96 h in the presence of the drugs as outlined in **(A)**. NK cell proliferation was quantified by gating on CD3^−^ CD56^+^ NK cells and definition of dividing eFluor 670^low^ NK cells. **(C)** PBMC of healthy donors (*n* = 4) were stimulated with K562 cells at E:T ratios of 1:1 or 3:1 for 24 h in the presence of the drugs as outlined in **(A)**. IFN-γ secretion was determined by the ELISpot technique where the number of IFN-γ-producing cells is defined as spot-forming cells counted by CTL ELISpot reader.

CD16-mediated degranulation was also significantly reduced in the presence of CsA, Tac, and Sir without additional IL-2 stimulation (Figure 7A). However, this inhibitory effect was completely abrogated in the presence of IL-2 confirming previous observations on NK cell activation with two NK cell lines, NKL and NK92 (data not shown). In contrast to degranulation, NK cell proliferation in response to K562 cells was not significantly reduced upon treatment with CsA, Tac, and Sir for 48 h (Figure 7B). The addition of MMF did neither affect degranulation nor proliferation of NK cells. After 96 h incubation, inhibition of NK cell proliferation could not be detected anymore indicating that the intrinsic cytokine production following K562-stimulation may counterbalance the initial inhibitory effect of the drugs.

In order to demonstrate the influence of CNI and mTORi on cytokine secretion, IFN-γ ELISpot assays were performed using K562 target cells as stimulus in the absence of IL-2. A strong inhibitory effect of CsA and Tac on IFN-γ secretion was observed in all three donors although with some variation in the sensitivity to the drugs (Figure 7C). The capability of Sir to reduce IFN-γ secretion varied substantially between donors, ranging from significant inhibition in donor 2 and 3 to basically no effect in donor 1 suggesting a donor-specific involvement of the mTOR signaling pathway in IFN-γ induction. In contrast, MMF treatment of PBMC had no impact on IFN-γ secretion in all three donors.

These striking differences between the two CNI regarding NK cell phenotype and function indicate that their suppressive mechanism in NK cells differs from the well-studied NFAT-based suppressive effect in T cells. Moreover, IL-2 receptor-mediated signals are able to override the suppressive effects of CNI and mTORi, especially in effector functions like degranulation that do not require transcriptional activity. In addition, CsA vs. Tac may impinge on the peripheral NK cell repertoire by the down-regulation of CD16, CD6, CD69, HLA-DR, and the simultaneous up-regulation of CD161, CD94/NKG2A which is likely to be associated also with functional alterations.

## Discussion

In our first analyses of peripheral NK cells from kidney recipients between 1 and 261 months after transplantation, we could demonstrate that the NK cell phenotype and repertoire differs significantly from healthy individuals without kidney transplantation and subsequent immunosuppressive treatment. The overall representation of NK cells within the lymphocyte compartment was not altered between KTx-patients in their first year after transplantation and later time points or compared to healthy individuals. However, the presence of donor-specific anti-HLA class I- and/or class II antibodies (DSA) in sera of KTx-patients was associated with lower absolute numbers of CD56^dim^ and CD56^bright^ NK cells in peripheral blood. The observation that the presence of MICA-specific antibodies in patient sera did not correlate with decreased NK cell numbers supports the assumption that donor-specificity represents a functional link between DSA and NK cells presumably by a recruitment of CD16^+^ NK cells into the graft and activation of ADCC mechanisms. DSA are strongly associated with chronic humoral rejection, one of the major causes for renal allograft dysfunction which is defined by a triad of DSA, C4d complement deposition in peritubular capillaries and transplant glomerulopathy (Farkash and Colvin, 2012). Since the association between serum DSA and C4d-mediated complement activation in kidney biopsies is imperfect, an involvement of NK cells was recently postulated primarily on the basis of animal models (Akiyoshi et al., 2012). In order to prove a direct involvement of NK cells in ABMR and renal dysfunction, it will be necessary to demonstrate the presence of CD16^+^ NK cells and their ADCC potential in kidney tissue. Expression profiling of kidney biopsies identified patterns of NK-related transcripts that were highly associated with DSA and ABMR (Hidalgo et al., 2010, 2012). Although the presence of MICA-specific antibodies was found to be associated with increased frequency of graft loss in a large cohort of KTx-patients (Zou et al., 2007), the role of MICA/B in renal transplantation is still discussed controversially (Cox et al., 2011). In our small cohort, no influence of MICA antibodies could be demonstrated on number or distribution of peripheral NK cells. Since the MICA receptor NKG2D is expressed by virtually all human NK cells and CD8^+^ T cells and co-regulates cytokine production (Barber and Sentman, 2011), MICA antibodies may also interfere with T cells and, therefore, not promote alterations in the NK cell repertoire.

Regarding the two major peripheral NK cell subsets, the CD56/16^bright^ NK cell subset was found to be overrepresented significantly in KTx-patients. Using the Trucount technique, the CD56/16^dim^ population could be subdivided into the classical CD56/16^dim^ NK cells and a new CD56/16^low^ subset characterized by a remarkably low CD56 and CD16 density. In the CD56/16^dim^ NK cell compartment, the alterations were primarily associated with a selective down-modulation of the Fcγ-RIII, CD16, and the scavenger receptor CD6 that was previously shown to characterize the most prominent peripheral NK cell subset (Braun et al., 2011). A transient decrease in CD16 expression on NK cells can be observed during the degranulation process following triggering of CD16 itself or other activating receptors like NCR (own unpublished observations). This degranulation-associated down-modulation of CD16 *in vitro* is mediated by internalization as well as by shedding via metalloproteases and hallmarks recently activated NK cells (Mota et al., 2004). However, in HIV-patients, low expression of CD16 on CD56^dim^ NK cells was shown to be associated with impaired ADCC indicating that decreased CD16 levels may also be associated with a functionally exhausted status of NK cells (Lichtfuss et al., 2011). In the context of stem cell transplantation, down-modulation of CD16 and KIR on NK cells following IL-2 or IL-15 stimulation in combination with CsA was shown to be accompanied by increased NKp30 and decreased NKp44 and NKG2D expression and reduced NFAT-dephosphorylation and nuclear translocation (Wang et al., 2007). In separated CD56^dim^ and CD56^bright^ NK cells of healthy donors, stimulation by IL-2 + IL-15 was only weakly suppressed by CsA compared to the mTORi rapamycin or MMF (Eissens et al., 2010). This discrepancy to our results may be due to major experimental differences to our *in vitro* setting by NK cell separation, drug dosage, and addition of IL-15 to the culture conditions. This interpretation is supported by the finding that NK cell stimulation by IL-12 or IL-18 in combination with CsA resulted in increased IFN-γ production suggesting that the CsA effect on NK cells may vary substantially according to the microenvironment. The differences in the individual responses to CNI may also be mediated by single-nucleotide polymorphisms (SNPs) that have been shown to either influence T cell function directly like a polymorphism in the IL-17 promoter (Espinoza et al., 2011) or indirectly by polymorphic variants of transcription factors such as the Foxo family (Hedrick et al., 2012). Since T and NK cells share several signaling pathways for cytokines and chemokines, these functionally relevant polymorphisms may also influence NK cell activity and response to calcineurin or mTOR inhibitors, respectively.

In AML-patients after SCT and GVHD treated with FK506 (Tac), NK cell activity in terms of cytotoxicity, cytokine secretion, and proliferation was found to be impaired. In addition, expression of NKG2D, CD48, and DNAM-1 as well as clustering of adhesion molecules such as CD2, CD58, and CD49d was found to be reduced (Kim et al., 2010). Since no alteration of DNAM-1 expression on NK cells could be observed in our kidney transplanted patient cohort (data not shown), it can be assumed that the disease context has substantial influence on the sensitivity of NK cell receptors toward immunosuppressive drugs. A dose- and time-dependent effect of CsA and Tac was demonstrated for KTx-patients whereby impaired NK activity coincided with increased viral infection (Morteau et al., 2010). Moreover, this study showed that conditioning of kidney recipients with alemtuzumab (anti-CD52) had only weak effects on NK cell distribution while basiliximab (anti-CD25) treatment is associated with alterations of the CD56^dim^/CD56^bright^ ratio. These findings support our observation that NK cells are sensitive to treatment with CNI even in different diseases. Nevertheless, the complex receptor expression and regulation of NK cells generates a lot of variables that have to be taken into account.

In our setting, detailed analyses of activation markers like CD25, CD69, and HLA-DR and NK receptors like CD161 and CD94/NKG2A revealed significant differences in these subsets indicating that the quality of the immunosuppressive treatment represented by different CNI and mTORi can impinge on the distribution of NK cell repertoires in peripheral blood. The different NK cell patterns with lower numbers of CD16^+^ and CD6^+^ CD56^dim^ NK cells and elevated CD16^−^ and CD6^−^ CD56^dim^ NK cells as well as CD6^−^ CD56^bright^ NK cell subsets under Tac-treatment may also reflect a different activation status of NK cells in this pharmacological constellation which may be due to a different availability of co-medication of immunosuppressive agents. For example, MMF undergoes an elevated enterohepatic circulation leading to higher mycophenolic acid (MPA) drug levels in combination with Tac whereas this effect is lacking in combination with CsA (Brown et al., 2002). Thus, it may be also possible that decreased activity of the inositol-mono-phosphate-dehydrogenase under combined therapy with Tac/MMF directly affects certain activation markers on NK cells, a phenomenon that was not studied so far (Devyatko et al., 2008).

Classical CNI, CsA, and Tac, both affect the NFAT-pathway by binding either to cyclophilin or to FKB12 and inhibiting the phosphatase activity of calcineurin which leads to prevention from dephosphorylation of the transcription factor NFAT, its nuclear translocation and NFAT-driven gene expression of IL-2, IFN-γ, GM-CSF, and others. In contrast, the involvement of the mTOR pathway is currently intensively for regulatory T cells (Treg) in comparison to effector T cells (Kim et al., 2010). While both pathways were studied primarily in T cells as primary target cells of CNI, only limited information was available regarding the effect of CNI vs. a combination with mTORi on the composition of NK cell subsets in patients after kidney transplantation. The observation that one patient who was initially treated with CsA for 5 months and “switched” later on to Tac due to a mild biopsy-proven rejection episode had maintained his initial CsA-driven NK cell pattern suggests that the onset of immunosuppression determines the alteration in the NK cell repertoire even when another CNI is applied afterward for months. This manifestation could be mediated by similar mechanisms involved also in Th1/Th2 differentiation like epigenetic regulation by promoter methylation or regulation of transcription factors, for instance (Lawson et al., 2012).

In summary, we could demonstrate that the type of immunosuppression has significant impact on the peripheral NK cell repertoire of kidney transplant recipients. This observation adds some novel aspects to the published studies on NK cell phenotype and function in the context of stem cell and organ transplantation showing that immunosuppressive drugs also affect NK cells at various levels. Substantial differences between stem cell and organ transplantation can be observed with respect to alterations in the composition of NK cell subsets or effector functions such as cytotoxicity, proliferation, and cytokine secretion under the influence of immunosuppression. This variability indicates an important role of the clinical context for the efficacy of immunosuppression on NK cells in peripheral blood. At least for kidney transplantation, the peripheral NK cell repertoire may represent an important hallmark of immunosuppression and NK cells may even be useful as sensors for a guided adjustment of immunosuppression in the future. However, the relevance of NK cells for the local immune response in the grafted tissue needs to be explored in future studies with biopsy material in addition to peripheral blood of kidney recipients.

## Conflict of Interest Statement

The authors declare that the research was conducted in the absence of any commercial or financial relationships that could be construed as a potential conflict of interest.

## Authors Contribution

Christine Neudoerfl has written large parts of the ms. and performed all *in vitro* analyses with NK cells under immunosuppression. Bernadett J. Mueller has performed flow cytometry stainings and analyses including statistics and graphics and contributed to writing of the ms. Cornelia Blume was responsible for patient recruitment, withdrawal of blood, generation of a clinical data base, selection of patients, and writing of the clinical parts of the ms. Kerstin Daemen, Maja Stevanovic-Meyer, and Jana Keil performed all flow cytometry staining experiments. Frank Lehner was responsible for transplantation and patient care including selection of patients for this study. Hermann Haller is responsible for the protocol biopsy program at MHH and thus, also for patient selection into this study. Christine S. Falk was responsible for the coordination of the study, quality control of experimental and clinical data, for writing of the ms, and serves as corresponding author.

## Abbreviations

ABMR, antibody-mediated rejection; CNI, calcineurin inhibitor; CsA, Cyclosporin A; DSA, donor-specific antibodies; HLA, human leukocyte antigen; IS, immunosuppression; KIR, killer-immunoglobulin-like receptors; KTx, kidney transplantation; MMF, mycophenolate mofetil; MPA, mycophenolic acid; mTORi, mammalian target of rapamycin (mTOR) inhibitor; NK cells, natural killer cells; Sir, sirolimus; Tac, Tacrolimus; Tx, transplantation.
